# Communicating tailored risk information of cancer treatment side effects: Only words or also numbers?

**DOI:** 10.1186/s12911-020-01296-7

**Published:** 2020-10-27

**Authors:** Ruben D. Vromans, Steffen C. Pauws, Nadine Bol, Lonneke V. van de Poll-Franse, Emiel J. Krahmer

**Affiliations:** 1 Department of Communication and Cognition, Tilburg Center for Cognition and Communication, Tilburg School of Humanities and Digital Sciences, Tilburg University, Warandelaan 2, 5037 AB Tilburg, The Netherlands; 2 Collaborative Care Solutions, Philips Research, Eindhoven, The Netherlands; 3 Department of Research and Development, Netherlands Comprehensive Cancer Organization (IKNL), Utrecht, The Netherlands; 4 Division of Psychosocial Research and Epidemiology, The Netherlands Cancer Institute, Amsterdam, The Netherlands; 5 Department of Medical and Clinical Psychology, Tilburg University, Tilburg, The Netherlands

**Keywords:** Tailoring, Personalization, Risk communication, Message format, Cancer, Treatment side effects, Patient information, Shared decision-making

## Abstract

**Background:**

The increased availability of patient reported outcome data makes it feasible to provide patients tailored risk information of cancer treatment side effects. However, it is unclear how such information influences patients’ risk interpretations compared to generic population-based risks, and which message format should be used to communicate such individualized statistics.

**Methods:**

A web-based experiment was conducted in which participants (*n* = 141) read a hypothetical treatment decision-making scenario about four side effect risks of adjuvant chemotherapy for advanced colon cancer. Participants were cancer patients or survivors who were recruited from an online Dutch cancer patient panel. All participants received two tailored risks (of which the reference class was based on their age, gender and tumor stage) and two generic risks conveying the likelihood of experiencing the side effects. The risks were presented either in words-only (‘common’ and ‘very common’), or in a combination of words *and* corresponding numerical estimates (‘common, 10 out of 100’ and ‘very common, 40 out of 100’). Participants’ estimation of the probability, accuracy of their estimation, and perceived likelihood of occurrence were primary outcomes. Perceived personal relevance and perceived uncertainty were secondary outcomes.

**Results:**

Tailored risks were estimated as higher and less accurate than generic risks, but only when they were presented in words; Such differences were not found in the verbal and numerical combined condition. Although tailoring risks did not impact participants’ perceived likelihood of occurrence, tailored risks were perceived as more personally relevant than generic risks in both message formats. Finally, tailored risks were perceived as less uncertain than generic risks, but only in the verbal-only condition.

**Conclusions:**

Considering current interest in the use of personalized decision aids for improving shared decision-making in oncology, it is important that clinicians consider *how* tailored risks of treatment side effects should be communicated to patients. We recommend both clinicians who communicate probability information during consultations, and decision aid developers, that verbal descriptors of tailored risks should be supported by numerical estimates of risks levels, to avoid overestimation of risks.

## Background

After a cancer diagnosis, most patients want to be fully informed about the possible treatment options and the associated risks of side effects to support a well-informed treatment decision-making process [1, 2]. For instance, colorectal cancer patients eligible for chemotherapy should be informed about the chances of experiencing adverse effects such as neuropathy or changes in smell and taste. Such risk statistics are typically communicated by the clinician during a consultation and/or incorporated into tools such as patient decision aids [3], and are therefore an essential part of shared decision-making [4]. However, patients often have difficulty understanding and interpreting risks [5], especially those patients with low numeracy of health literacy skills [6], which can further influence treatment decision-making [7, 8]. Due to advances in artificial intelligence and personalized medicine, there has been rapid growth in the development of tailored risk communication tools in cancer care [9–12], with the aim to provide patient’s risk information about treatment side effects based on their personal clinical and sociodemographic characteristics. Despite great promise of such individualized data-driven tools [13], it is unclear whether (1) tailored risks influence risk estimates and perceptions and lead to more or less accurate risk estimates compared to generic risks, and (2) which message format should be used to communicate such individualized statistics to patients.

Typically, risk information about possible treatment side effects is generic and mostly based on the “average patient”, such as information presented in randomized controlled trials or patient reported outcome reports [14]. Such average statistics make it hard to relate outcomes to individual patients [15], particularly because they often do not contain a clear description of to whom the risk estimates refer (i.e., the reference class) and may therefore be a factor in the misunderstanding of the risk information about treatment side effects [16]. *Tailoring* risk information of side effects adjusted to the clinical (e.g., tumor stage) and sociodemographic (e.g., age, gender) characteristics of an individual patient may increase the perceived personal relevance of risk information, thereby increasing the likelihood that patients will process the tailored information with more deliberation, consideration, and evaluation [17, 18]. In fact, several studies have shown that tailored risk estimates may improve the accuracy of patients’ estimations of probabilities and may increase their perceived likelihood of occurrence in both the general health context [19] as well as in the domain of cancer risk and screening [20, 21]. Therefore, tailoring side effect risks may be an effective communication strategy for enhancing the accuracy of patients’ risk estimates and for increasing risk perceptions.

An important consideration for clinicians, health educators and patient tool developers is through which *message format* they should communicate tailored risk statistics, using for instance verbal and/or numerical formats [15]. Verbal risks can be expressed via descriptions such as rare, likely, or very common. The European Commission provided guidelines on using particular verbal descriptors associated with corresponding numerical estimates (Table 1) [22]. The problem is that such phrases are often interpreted in different ways by different patients, typically causing overestimations of the actual occurrence of the side effect [23–26]. Another way of communicating risks is through combining verbal information with numerical estimates, such as percentages, probabilities, or natural frequencies [22, 26]. Although experimental studies have consistently shown that a combination of verbal and numerical formats of generic risks are estimated as lower and perceived as less likely to occur than verbal descriptions alone [23–27], it is not known to what extent such results apply to tailored risks. It is important to study this, as recent studies suggest that verbal risk labels without accompanying numerical information are still frequently used by oncologists [28] or incorporated in patient decision aids for communicating tailored risks of treatment side effects [29–31].

**Table 1:** Verbal descriptors of side effects risks and their corresponding numerical probabilities as recommended by the European Commission [22]

Verbal descriptor	Corresponding numerical frequency interval
Very common	May affect more than 1 in 10 people (≥ 1/10)
Common	May affect up to 1 in 10 people (≥ 1/100 to < 1/10)
Uncommon	May affect up to 1 in 100 people (≥ 1/1000 to < 1/100)
Rare	May affect up to 1 in 1,000 people (≥ 1/10,000 to < 1/1000)
Very rare	May affect up to 1 in 10,000 people (< 1/10,000)
Not known	Frequency cannot be estimated from the available data

### Present study and hypotheses

In the present study, we will examine the impact of tailoring (tailored vs. generic risks) and message format (verbal-only vs. verbal and numerical combined format) of risks of cancer treatment side effects on cancer patients’ risk interpretations. We will use estimation of probability, accuracy of estimation of probability, and perceived likelihood of occurrence as primary outcome variables. First, regarding the influence of tailoring, we expect that risks that are tailored will be perceived as more likely to occur than generic risks [20, 32].

#### **H1:**

Compared to generic risks of treatment side effects, tailored risks will be perceived as more likely to occur.

Second, given the growing importance of replication research in the empirical sciences for improving the reproducibility of earlier study’s results [33], we attempt to conceptually replicate previous findings on the effect of message format on peoples’ risk interpretations. Previous studies have consistently shown that people viewing generic risk information in a verbal-only format estimate the probability as higher and less accurate [23, 25], and perceive these risks as more likely to occur than people viewing risks in a verbal and numerical combined format [23–26]. We expect this impact of message format to persist for tailored risks as well.

#### **H2:**

 Compared to risks of treatment side effects presented in a verbal and numerical combined format, risks presented in a verbal-only format will be estimated as (a) higher) and (b) less accurate, and (c) perceived as more likely to occur.

Third, regarding the combined effect of tailoring and message format, we assume that tailored risks expressed as words and numbers combined should improve peoples’ estimated risk accuracy even more compared to generic risk information [19–21]. This is because especially in this situation, people should have less reason to deviate from the actual tailored risk statistic being communicated.

#### **H3:**

 Compared to generic risks of treatment side effects, tailored risks will be estimated as more accurate than generic risks, but only when the risks are presented in a verbal and numerical combined format.

Finally, we will assess perceived personal relevance and perceived uncertainty of the risk information as secondary outcome measures, for which we propose the following two hypotheses:

#### **H4:**

 Tailored risks of treatment side effects will be perceived as more personally relevant than generic risks, regardless of the message format.

#### **H5:**

 Tailored risks of treatment side effects will be perceived as less uncertain than generic risks, regardless of the message format.

## Methods

### Study design

We used a 2 (tailoring: tailored vs. generic) × 2 (message format: verbal-only vs. verbal and numerical combined) × 2 (probability rate: low vs. high) mixed design, with repeated measures on the first and third factor. Participants were randomly assigned to one of the two message format conditions. We included probability rate as a methodological variable to investigate whether the effects of tailoring and message format are similar for high and low probability rates ^25^. We used estimation of probability, accuracy of estimation of probability, and perceived likelihood of occurrence as primary outcome variables, and perceived personal relevance and perceived uncertainty as secondary outcome variables.

### Participants

Native Dutch adults between the ages of 18 and 70 who had been diagnosed with cancer in the past were selected from the scientific panel of the online cancer community platform Kanker.nl to participate in our study. We selected people who had been in a similar health situation before, since they are better able to imagine the given scenarios (compared to, for instance, a student sample), thus enhancing the generalizability of our results [34]. Patients who were diagnosed with colorectal cancer in the past were excluded from participation due to prior personal experience. As part of the pre-registered analysis (https://osf.io/ygchx), power calculations were conducted prior to data collection to determine our sample size using the program G*Power 3.1 [35]. Previous meta-analyses have indicated small effect sizes for tailoring effects on perceived likelihood of occurrence [36], and medium effect sizes for message format effects on estimation of probability and perceived likelihood of occurrence [23]. To detect a small effect (effect size *f* = 0.10) with a 2 × 2 × 2 mixed design, a sample of 136 participants was needed (power = 0.8, alpha = 0.05). We therefore aimed for a minimum of 136 participants.

### Stimulus materials

All participants received two tailored and two generic risk statistics for the occurrence of four possible side effects after adjuvant chemotherapy including fatigue, neuropathy, taste and smell changes, and diarrhea, respectively. Tailoring was established by manipulating the *reference class* (i.e., denominator) to which the risk statistic applies. More specifically, tailored risks contained a reference class based on participants’ reported gender (male or female), age group (in 5-year bins between 15 and 69 years), and tumor stage (advanced colon cancer as stated in the scenario). For example: ‘This side effect is common (occurs in 10 out 100 men like you, aged between 65 and 69 years with advanced colon cancer)” (Table 2). Generic risks descriptions were fixed and included a reference class that was not tailored toward patient and tumor characteristics. For example: “This side effect is common (occurs in 10 out of 100 people)” (Table 2).

**Table 2:** Development and structure of the risk information about the likelihood of occurrence for each experimental condition

	Verbal-only condition		Verbal and numerical combined condition	
	Generic	Tailored	Generic	Tailored
Low probability rate	This side effect is common	This side effect is common in [gender] like you, aged between [age group] years with advanced colon cancer	This side effect is common (occurs in 10 out of 100 people)	This side effect is common (occurs in 10 out of 100 [gender] like you, aged between [age group] with advanced colon cancer)
High probability rate	This side effect is very common	This side effect is very common in [gender] like you, aged between [age group] with advanced colon cancer	This side effect is very common (occurs in 40 out of 100 people)	This side effect is very common (occurs in 40 out of 100 [gender] like you, aged between [age group] with advanced colon cancer)

The risk information was presented in Dutch to the participants

Half of the participants received the risk only in words (verbal-only condition), and the other half in a combination of words and numbers (verbal and numerical combined condition). Within the verbal-only condition, we selected the verbal descriptors ‘common’ (*vaak* in Dutch) for representing a low probability rate and ‘very common’ (*zeer vaak* in Dutch) for representing a high probability rate. Following the recommendations proposed by the European Commission, we used the corresponding natural frequency estimates ‘10 out of 100’ for representing a low probability rate and ‘40 out of 100’ for representing a high probability rate [3, 5, 15, 22]. To exclude the possible effect that a specific side effect could influence higher risk estimates, the combination of tailoring, probability rate and type of side effect was randomized, as well as the order of tailored and generic risks in combination with the probability rate.

### Procedure

Data collection took place in May 2019. A representative of Kanker.nl sent a link of our web-based experiment to participants of the cancer patient panel. When entering the online experiment, an introductory text was shown, followed by questions on background and medical characteristics. The reported gender and age group were subsequently used for tailoring the reference class of the tailored risk information. Participants then read a short scenario in which they imagined being diagnosed with advanced colon cancer and discussing adjuvant chemotherapy as a treatment option with their doctor. We chose colon cancer as the disease context because both men and women can be diagnosed with this form of cancer (versus, for example, prostate cancer). This allowed us to include gender as a tailoring factor of the risk information. Participants were told that they were receiving a decision aid from their doctor including information about four possible side effects after adjuvant chemotherapy. Each description consisted of three elements: the name of the side effect, a short description of the side effect, and risk information about the likelihood of experiencing the side effect. This was followed by the assessment of the primary and secondary outcome measures. In the final part of the experiment, we measured participants’ subjective numeracy skills and prior history with chemotherapy and/or one of four mentioned the side effects. Participants were then debriefed about the main purpose of the experiment and thanked for their participation.

### Measures

#### Primary outcome measures

We had three primary outcome measures for measuring risk interpretations, based on the meta-analysis by Büchter and colleagues [23] and the studies by Knapp and colleagues that we attempted to replicate [24, 25]. First, *estimation of probability* was assessed using the question “What do you think is the probability **you** will experience this side effect”, measured as a percentage between 0 and 100 [24]. Second, the *accuracy of the estimation of probability* was determined by computing the absolute difference between the actual risk of each side effect occurring and each participant’s estimated risk of that side effect occurring. Scores closer to zero were therefore more accurate (for similar reasoning, see [21, 25]). Third, *perceived likelihood of occurrence* was assessed using the question “How likely is it that **you** will experience this side effect?”, measured on a 6-point scale, with 1 as ‘not likely at all’ and 6 as ‘very likely’ [23, 24].

#### Secondary outcome measures

We also included two secondary outcome variables. First, *perceived personal relevance* was assessed using the items “The risk information about the side effect was made personally for me” and “The way how the risk information was being presented was relevant to me” (measured on a 5-point scale, with 1 as ‘strongly disagree’ and 5 as ‘strongly agree’) [32]. Second, *perceived uncertainty* was assessed by asking the question “How uncertain do you think is this likelihood of experiencing this side effect after chemotherapy?”, measured on a 6-point scale, with 1 as ‘not at all’ and 6 as ‘extremely’ [37].

#### Individual difference measures

Individual differences in subjective numeracy were assessed by the Subjective Numeracy Scale (SNS [38]), which is an 8-item self-assessment for determining participants’ quantitative ability and preferences for receiving numerical information (measured on a 6-point scale, with 1 as ‘least numerate’ and 6 as ‘most numerate’). The SNS has proven to be a valid and reliable measure, and correlates strongly with objective numeracy measures [39]. For the current study, we used the Dutch version of the SNS [40]. The mean subjective numeracy score was determined by computing the average score of the eight items.

### Statistical analyses

We conducted a 2 (within-subjects: tailoring) × 2 (between-subjects: message format) × 2 (within-subjects: probability rate) mixed-model multivariate analysis of variance (MANOVA).^1^ The dependent variables were our three primary outcome measures; estimation of probability, accuracy of estimation of probability, and perceived likelihood of occurrence (see Additional file 1 for full results). If applicable, significant interaction effects were further analyzed by means of simple effect analyses. As an additional exploratory analysis, we controlled for individual differences by conducting a separate mixed-model multivariate analysis of covariance (MANCOVA) with subjective numeracy skills and prior history with chemotherapy and/or one of the side effects as covariates. For this exploratory analysis, only results that deviate from the pre-registered MANOVA analysis were reported (Additional file 1). For our two secondary outcome measures, we conducted two separate mixed-model ANOVAs, with repeated measures on the first and third factor. The dependent variables were perceived personal relevance and perceived uncertainty. Data on patient and tumor characteristics for the two message format conditions were compared using chi-square tests for categorical variables and t-tests for continuous variables. All statistical analyses were performed using SPSS version 24.0 (IBM Corporation, Somers, NY, USA). Tests were two-sided and considered statistically significant at *p* < .05. The study design, hypotheses, and analysis plan were pre-registered prior to data collection and analysis within the Open Science Framework (https://osf.io/j74dt/). Ethical approval was granted by the Research Ethics and Data Management Committee of the Tilburg School of Humanities and Digital Sciences of Tilburg University (ID REDC.2019.26).

## Results

### Participants

**Fig. 1:**
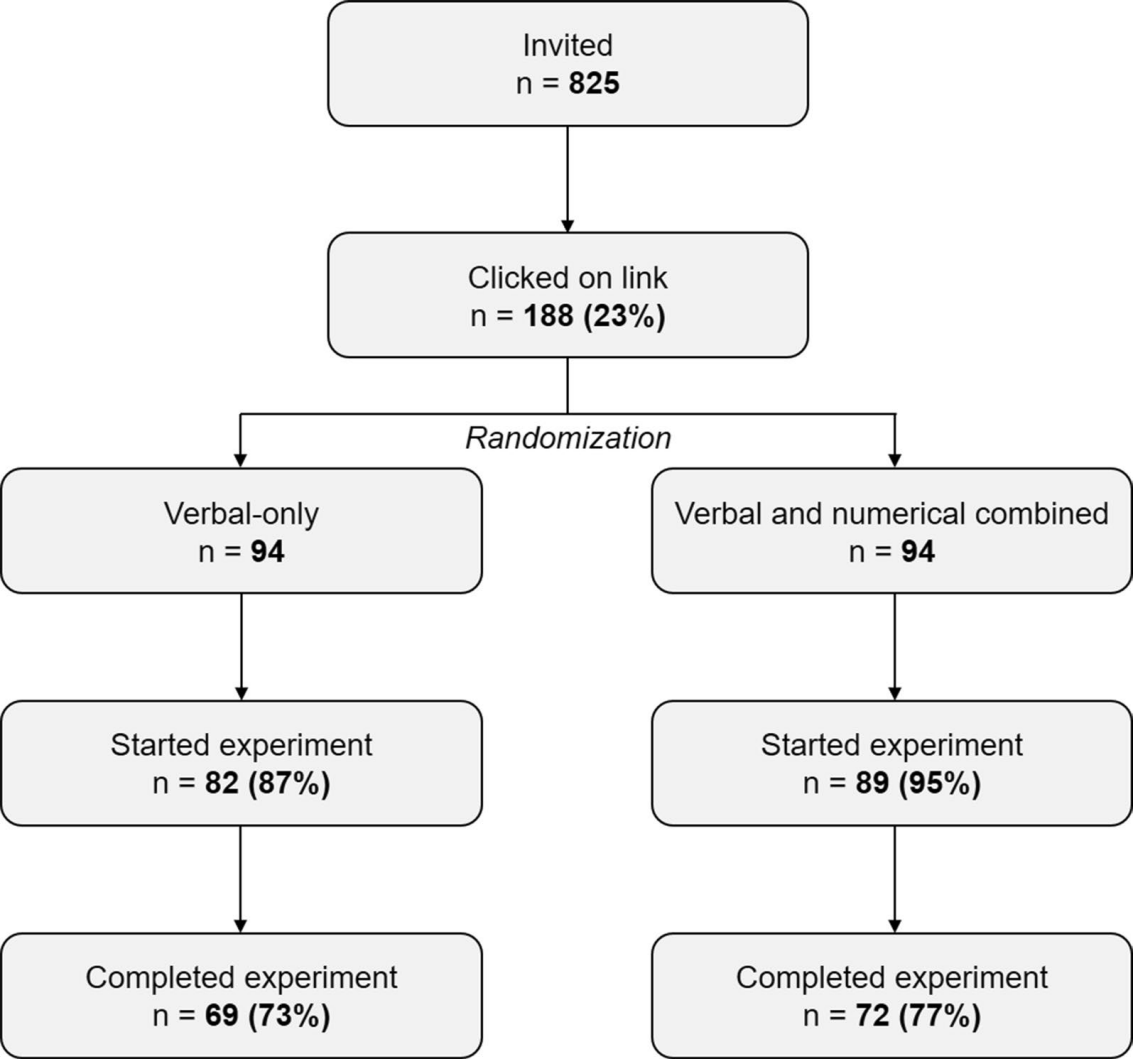
Flowchart of the data collection process

Out of 825 people who were invited to participate, 188 (23%) clicked the link to launch the survey. Of those, 171 (91%) continued beyond the informed consent page, and 141 (75%) fully completed the survey (Fig. 1). All completed cases were analyzed. Completion rates were consistent across experimental conditions (73% in the verbal-only condition, 77% in the verbal and numerical combined condition). The mean age of participants was 57.3 years (*SD* = 7.4), and the participants in both message format conditions were comparable in terms of sociodemographic and disease-related characteristics (all *p* values > .10, Table 3).

**Table 3:** Participant characteristics by message format condition

	Verbal-only (*n* = 69)		Verbal and numerical combined (*n* = 72)		*p*
	*n*	%	*n*	%	
Gender					
Female	45	35	46	36	
Male	24	65	26	64	.869
Age at time of experiment, mean (SD)	57.72 (7.29)		56.83 (7.50)		.469
< 50 years	8	11	13	18	
50–65 years	44	64	49	68	
> 65 years	17	25	10	14	.201
Education					
Primary school	8	11	12	17	
Secondary school	17	25	24	33	
College/University	44	64	36	50	.255
Tumor					
Breast	22	32	22	31	
Hematological^a^	13	19	8	12	
Urological^b^	10	15	13	18	
Gynecological^c^	7	10	10	14	
Head and neck	4	6	6	8	
Lung	2	3	4	5	
Skin	3	4	2	3	
Gastroenterological^d^	3	4	2	3	
Other^e^	3	4	4	5	
Unknown	2	3	1	1	.912
Years since diagnosis, mean (SD)	6.48 (6.89)		5.01 (4.22)		.133
0–5 years	43	62	52	72	
6–10 years	12	17	13	18	
11–15 years	8	12	5	7	
> 15 years	6	9	2	3	.318
Treatment(s)					
Surgery	48	70	48	67	.712
Radiotherapy	40	58	35	49	.266
Chemotherapy	40	58	37	51	.433
Immunotherapy	13	19	11	15	.574
Hormone therapy	18	26	24	33	.347
Other	16	23	13	18	.451
Prior experience side effects					
Fatigue	55	80	49	68	.116
Neuropathy	29	42	26	36	.471
Smell and taste changes	30	44	27	38	.470
Diarrhea	14	20	11	15	.436
None of the above	7	10	9	13	.659
Subjective numeracy, mean (SD)^f^	4.51 (0.81)		4.64 (0.86)		.384

^a^Lymphoma, Leukemia, Multiple myeloma

^b^Prostate, bladder

^c^Uterus, cervix, ovary

^d^Esophageal, anus, GIST, gall bladder, but excluding colorectal cancer

^e^Brain, renal cell, undifferentiated pleomorphic sarcoma, neuroendocrine tumor

^f^α = .82; *SD* standard deviation

### Effects on primary outcome measures

In both message format conditions, participants’ estimated probabilities strongly correlated with the accuracy of their estimated probabilities (*r*_verbal-only_ = − .984, *p* < .001, *r*_verbal+numerical_ = − .943, *p* < .001) and perceived likelihood of occurrence (*r*_verbal-only_ = .820, *p* < .001, *r*_verbal+numerical_ = .738, *p* < .001), which, in turn, strongly correlated with participants’ accuracy of estimated probabilities (*r*_verbal-only =_ -.813, *p* < .001, *r*_verbal+numerical_ = .728, *p* < .001).

#### Effects of tailoring

There was a significant main effect of tailoring on the estimation of probabilities and accuracy of estimation of probabilities. Tailored risks were estimated as higher, *F*(1, 125) = 6.25, *p* = .023, η_p_^2^ = .04, and less accurate, *F*(1, 125) = 6.25, *p* = .014, η_p_^2^ = .05, than generic risks (Tables 4, 5). However, in contrast to our hypothesis (H1), there was no significant main effect of tailoring on the perceived likelihood of occurrence, indicating that tailored risks were not perceived as more likely to occur than generic risks, *F*(1, 125) = 1.79, *p* = .183, η_p_^2^ = .01. It should be noted that these tailoring effects were not found when controlling for individual differences in numeracy and prior history with the side effects (Additional file 1). Overall, the effects of tailoring did not depend on the probability rate (all *F*s < 1).

**Table 4:** Participants’ mean scores (with standard deviations within parentheses) on the primary and secondary outcome measures as a function of tailoring (tailored vs. generic risks) and message format (verbal-only vs. verbal and numerical combined) for low probability rate risks

Measures	Verbal-only “common”			Verbal and numerical combined “common, 10 out of 100”		
	Generic	Tailored	Total	Generic	Tailored	Total
*Primary measures*						
Estimation of probability^1^ (in %)	64.8 (20.1)	70.1 (19.6)^a^**	67.5 (19.8)	34.3 (29.9)	32.9 (30.8)	33.6 (30.4)^c^***
Accuracy of estimation of probability^2^ (in %)	54.8 (20.1)	60.1 (19.6)^a^***	57.5 (19.9)	24.9 (29.4)	23.7 (30.2)	24.3 (29.7)^c^***
Perceived likelihood of occurrence^3^	4.41 (1.09)	4.46 (1.01)	4.49 (1.05)	3.25 (1.52)	3.23 (1.57)	3.24 (1.55)^c^***
*Secondary measures*						
Perceived personal relevance^4^	3.16 (0.74)	3.46 (0.81)^a^***	3.34 (0.78)	3.19 (0.97)	3.40 (0.91)^b^***	3.30 (0.94)
Perceived uncertainty^5^	2.90 (1.19)	2.59 (1.19)^a^*	2.75 (1.19)	3.13 (1.50)	3.31 (1.47)	3.18 (1.49)

^1^“What do you think is the probability you will experience this side effect” (percentage between 0 and 100%)

^2^The absolute difference between the actual risk of each side effect occurring and each participant’s estimated risk (scores closer to zero are more accurate)

^3^“How likely is it that you will experience this side effect?” (1 = not likely at all, 6 = very likely)

^4^“The risk information about the side effect was made personally for me” and “The way how the risk information was being presented was relevant to me” (1 = strongly disagree, 5 = strongly agree, α = .87)

^5^“How uncertain do you think is this likelihood of experiencing this side effect after chemotherapy?” (1 = not at all, 6 = extremely)

^a^Mean differs significantly compared to generic risk within verbal-only risk condition

^b^Mean differs significantly compared to generic risk within verbal and numerical combined condition

^c^Mean differs significantly compared to total verbal-only risk; *p < .01, **p = .001, ***p < .001

**Table 5:** Participants’ mean scores (with standard deviations within parentheses) on the primary and secondary outcome measures as a function of tailoring (tailored vs. generic risks) and message format (verbal-only vs. verbal and numerical combined) for high probability rate risks

Measures	Verbal-only “very common”			Verbal and numerical combined “very common, 40 out of 100”		
	Generic	Tailored	Total	Generic	Tailored	Total
*Primary measures*						
Estimation of probability (in %)	71.0 (21.1)	78.9 (18.1)^a^**	74.9 (19.1)	53.5 (22.3)	54.0 (21.9)	53.7 (22.1)^c^***
Accuracy of estimation of probability (in %)	32.1 (19.5)	39.6 (16.7) ^a***^	35.8 (18.1)	17.4 (19.3)	17.9 (18.7)	17.7 (19.0)^c***^
Perceived likelihood of occurrence	4.73 (1.07)	5.02 (0.98)	4.87 (1.03)	4.23 (1.22)	4.28 (1.21)	4.26 (1.22)^c^***
*Secondary measures*						
Perceived personal relevance	3.16 (0.74)	3.47 (0.80) ^a^***	3.39 (0.77)	3.34 (0.89)	3.47 (0.86) ^b^***	3.40 (0.88)
Perceived uncertainty	2.55 (1.30)	2.41 (1.37)^a^*	2.48 (1.34)	2.73 (1.20)	2.91 (1.36)	2.82 (1.28)

^a^Mean differs significantly compared to generic risk within verbal-only risk condition

^b^Mean differs significantly compared to generic risk within verbal and numerical combined condition

^c^Mean differs significantly compared to total verbal-only risk; *p < .01, **p = .001, ***p < .001

#### Effects of message format

As hypothesized, there was a significant main effect of message format on the estimation of probabilities, *F*(1, 125) = 69.82, *p* < .001, η_p_^2^ = .36, accuracy of estimation of probabilities, *F*(1, 125) = 64.26, *p* < .001, η_p_^2^ = .34, and perceived likelihood of occurrence, *F*(1, 125) = 30.27, *p* < .001, η_p_^2^ = .20. The results therefore suggest that risks presented in a verbal-only format were estimated as higher (H2a), less accurate (H2b), and perceived as more likely to occur (H2c) than risks presented in a verbal and numerical combined format. These message format effects were also found when controlling for individual differences (all *p*s < .001; Additional file 1), and were more pronounced for low probability rates (all *p*s < .001).

#### Interaction effects between tailoring and message format

**Fig. 2:**
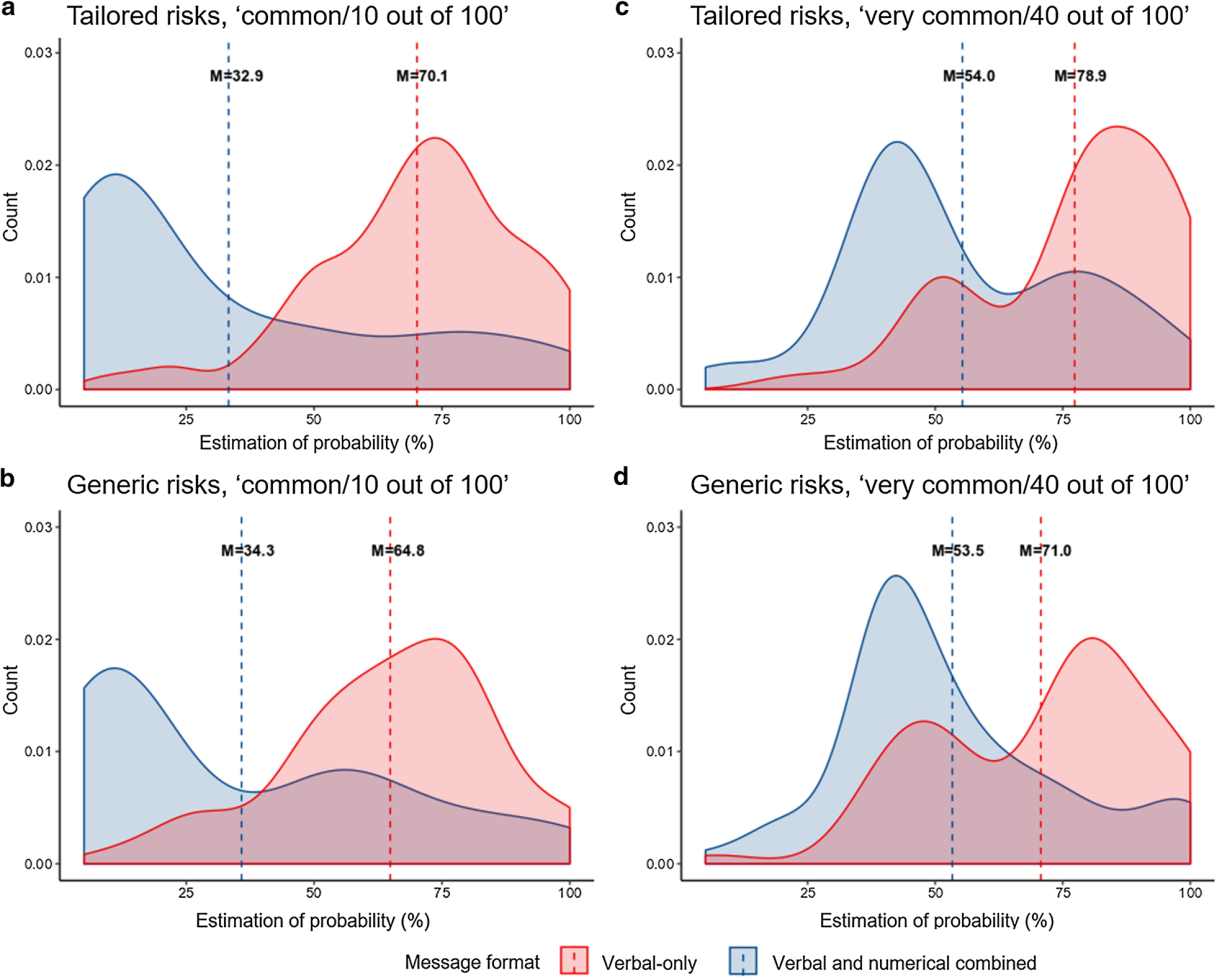
Comparisons of distribution of estimations of probabilities between verbal-only (red) and verbal and numerical combined (blue) message formats for **a** low probability tailored risks and **b** low probability generic risks, and for **c** high probability tailored risks and **d** high probability generic risks. The dotted lines represent the average estimated risks

There was a significant interaction effect between tailoring and message format on the accuracy of estimation of probabilities, *F*(1, 125) = 7.82, *p* = .006, η_p_^2^ = .06. Simple effect analysis showed that tailored risks were estimated as less accurate than generic risks in the verbal-only condition, (*p* < .001), but not in the combined condition (*p* = .833). This is in contrast to our hypothesis (H3), for which we expected tailored risks to be estimated as more accurate compared to generic risks, but only when expressed as words and numbers combined. There was also a significant interaction effect on the estimation of probabilities, *F*(1, 125) = 7.21, *p* = .008, η_p_^2^ = .06. Simple effect analysis revealed that tailored risks were estimated as higher than generic risks in the verbal-only condition (*p* = .001), but not in the combined condition (*p* = .789). Overall, these significant interaction effects were found for both probability rates, and when controlling for individual differences (Additional file 1). Finally, there was no significant interaction effect between tailoring and message format on perceived likelihood of occurrence, *F*(1, 125) = 1.79, *p* = .183, η_p_^2^ = .01. Figure 2 displays the distribution of estimations of probabilities (and the mean estimates) given by participants for each experimental condition.

### Effects on secondary outcome measures

As hypothesized (H4), participants perceived tailored risks as more personally relevant than generic risk information about side effects, *F*(1, 123) = 19.11, *p* < .001, η_p_^2^ = .13 (Tables 4, 5). This effect of tailoring occurred regardless of message format conditions, *F*(1, 123) = 2.36, *p* = .127, η_p_^2^ = .02, and probability rate, *F* < 1. Regarding perceived uncertainty, there was a significant interaction effect between tailoring and message format, *F*(1, 113) = 6.23, *p* = .014, η_p_^2^ = .05. Simple effects analysis showed that tailored risks in the verbal-only condition were perceived as less uncertain than generic risks (*p* = .007), but not in the verbal and numerical combined condition (*p* = .436), which partly confirms H5. Finally, risks with low probability rates were perceived as more uncertain than risks with high probability rates, *F*(1, 113) = 11.01, *p* < .001, η_p_^2^ = .09.

## Discussion

### Main findings

The current study demonstrates that message format matters when communicating tailored risk information of treatment side effects. We found that communicating tailored side effect risks leads to higher and less accurate risk estimates compared to generic risks, but only when the risks were communicated using words-only. Such differences were not found in the combined verbal and numerical condition. This suggests that communicating about side effect risks in words-only allows patients to overestimate and even inaccurately estimate their tailored risks [15, 41, 42]. Moreover, patients may take these individualized verbal risk labels as too personal, which in turn may lead to overestimations of the risks. However, these tailoring effects could not be found for perceived likelihood of occurrence, which may underscore that increases in risk estimations do not necessarily translate into increases in perceived likelihood of occurrence. Furthermore, we replicated the message format effect for Dutch verbal risk labels. More specifically, we showed that risks presented in a verbal-only format are estimated as higher and less accurate, and perceived as more likely to occur than risks presented in a combined verbal and numerical format [23, 25, 26].

However, tailored risks in a verbal and numerical combined format did not lead to more accurate risk estimates compared generic numerical risk information [19–21]. A possible explanation for this might be that the tailored and generic risks were shown separately and did not contain any comparative risk information. As a result, patients could not see their own risk score for a particular side effect in comparison with scores of other patients, especially for determining whether they were above or below average [43, 44]. Although there is currently a debate about whether comparative risk information should be provided to patients [45, 46], such communication strategy could improve people’s estimations of probabilities and perceived likelihood of occurrence in the context of tailored versus generic risks of side effects [19].

Finally, in both message formats, tailored risks are perceived as more personally relevant than generic risks, which is in line with past studies on tailoring effects in health communication [32]. In addition, this shows that by manipulating the reference class of probability outcomes our manipulation of tailoring was successful. We further found that when risks were presented only by means of verbal descriptors, tailored risks were perceived as less uncertain than generic risks. This suggests that tailored risks in the verbal-only condition were estimated as higher, and therefore perceived as more certain to occur.

### Limitations and suggestions for future research

A first limitation is that the research design uses a hypothetical decision-making scenario instead of a real decision-making scenario. To partially compensate for this, our sample consisted of cancer patients and survivors who were recruited from a Dutch cancer patient panel. Often, scenario-based experimental studies on effective risk communication strategies are conducted in student samples (for an overview, see [18]), who may not be familiar with a medical decision-making situation and may have different perceptions of risks and probability information about cancer [23, 47]. Although the use of cancer patients in our experiment contributed to the ecological validity of the results, future research to confirm our findings in a real-world treatment decision-making situation would be advisable.

Another limitation is that we tailored the risks based on a limited number of patient characteristics in a non-interactive way, to keep the experiment manageable and the results generalizable. Clinical prediction models in oncology settings typically utilize a larger variety of patient and tumor characteristics in decision-making (e.g., TNM-stage, the specific use of chemotherapy, or comorbidities) that is more extensive than we have dealt with in our study. Using such an interactive prediction modelling tool in which participants can enter their own personal and disease-related characteristics and see the impact of each characteristic on their personal risk could influence patients’ risk perception [48]. Despite this limitation, the tailored risks in our study were perceived as more personally relevant compared to the generic, population-based risks.

Finally, we only compared risks communicated through words or a combination of words and numbers, and did not consider the potential added value of visual aids as another message format. A plethora of research suggests that visual aids may increase understanding and perception of risk information [3, 15, 42, 49, 50]. For instance, bar charts may help to display the distinction between tailored and generic risks, and pictographs may communicate the number of people with similar characteristics that may experience the side effect compared to the number of people from the general population [15]. Therefore, it is suggested to investigate the impact of tailored risks through visually presented information compared to, for instance, numerical descriptions of risks.

### Implications

Despite these limitations, our findings have implications for research and practice. First, in line with guidelines and best practices for communicating complex medical data and risks in daily clinical practice and patient decision aids [3, 15, 42, 51], our results offer support for the recommendation to avoid verbal descriptions without numbers since they may lead to inaccurate risk estimates. Our findings suggest that this recommendation may become even more relevant when the risks are tailored and adjusted to sociodemographic and clinical characteristics of patients. This finding is useful for clinicians who discuss risks, health data, and other probability information during consultations in general with their patients and relatives, and especially for clinicians who are using modern decision-support systems (e.g., clinical prediction models) for estimating and communicating individualized treatment outcomes to patients. In addition, in light of the growing emphasis of personalized medicine [52], shared decision-making [4, 53], and the promising approaches of the delivery of tailored risk information through patient-centered decision aids [9–12], our results contribute to the empirical evidence on how best to communicate tailored risks to individual patients [54, 55].

## Conclusion

When communicating tailored risk information of treatment side effect to patients, using a combination of words and numbers will lead to more accurate risk estimates than when using words only. Although we found no evidence that tailoring of numerical risks leads to even more accurate risk estimates, doing so with verbal labels alone may have a negative impact on patients’ (accuracy) of estimation of risks. Given the strong movements toward personalized medicine and patient-centered healthcare, future research will have to determine whether other ways of presenting tailored risk information, such as comparative risk information or visual aids promote effective communication of tailored risks during cancer treatment decision-making.

## Supplementary information

**Additional file 1:** MANOVA and MANCOVA outcomes.

## Data Availability

The data that support the findings of this study can be accessed via https://osf.io/j74dt/.

## Footnotes

1 According to our pre-registration (https://osf.io/j74dt/), our main analysis consisted of a 2 (tailoring: tailored, generic) × 2 (message format: verbal-only, verbal and numerical combined) mixed-model multivariate analysis of variance (MANOVA) with repeated measures on the first factor. However, we decided to include probability rate as a methodological variable in our study design, which resulted in a 2 × 2 × 2 mixed-model MANOVA. This MANOVA was initially stated as an exploratory analysis in our pre-registration, but has now become the main analysis in this study.
