# Supplementary material for: Communicating tailored risk information of cancer treatment side effects: Only words or also numbers?
Source: BMC Med Inform Decis Mak. 2020 Oct 27;20:277. doi: 10.1186/s12911-020-01296-7 (PMC7590680; doi:10.1186/s12911-020-01296-7)
Supplement: Supplementary file 1 — Additional file 1: MANOVA and MANCOVA outcomes. [file 12911_2020_1296_MOESM1_ESM.docx]

**Additional file 1**

**Title:** MANOVA and MANCOVA outcomes

**Description:** For our main analysis, we conducted a 2 (within-subjects: tailoring) × 2 (between-subjects: message format) × 2 (within-subjects: probability rate) mixed-model multivariate analysis of variance (MANOVA)^1^. The dependent variables were our three primary outcome measures; estimation of probability, accuracy of estimation of probability, and perceived likelihood of occurrence. If applicable, significant interaction effects were further analyzed by means of simple effect analyses. As an exploratory analysis, we controlled for individual differences by conducting a separate 2 (within-subjects: tailoring) × 2 (between-subjects: message format) × 2 (within-subjects: probability rate) mixed-model multivariate analysis of covariance (MANCOVA) with subjective numeracy skills and prior history with chemotherapy and/or one of the side effects as covariates. The dependent variables were estimation of probability, accuracy of estimation of probability, and perceived likelihood of occurrence.

**Table A1.** Main and interaction effects on primary outcome measures (i.e., risk estimates, risk accuracy, and risk perception), resulting from a 2 (tailoring: tailored, generic) × 2 (message format: verbal-only, verbal and numerical combined) × 2 (probability rate: low, high) mixed-model MANOVA with repeated measures on the first and third factor.

|  | Primary outcome variables | | | | | | | | | | |
| --- | --- | --- | --- | --- | --- | --- | --- | --- | --- | --- | --- |
|  | Estimation of probability | | |  | Accuracy of estimation of probability | | |  | Perceived likelihood of occurrence | | |
| Main/Interaction Effects | *F* | *p* | η_p_^2^ |  | *F* | *p* | η_p_^2^ |  | *F* | *p* | η_p_^2^ |
| Tailoring | 5.32 | **.023** | .041 |  | 6.25 | **.014** | .048 |  | 2.40 | .124 | .019 |
| Message Format | 69.82 | **<.001** | .358 |  | 64.26 | **<.001** | .340 |  | 30.27 | **<.001** | .195 |
| Probability Rate | 72.71 | **<.001** | .368 |  | 93.59 | **<.001** | .428 |  | 65.86 | **<.001** | .342 |
| Tailoring × Message Format | 7.21 | **.008** | .055 |  | 7.82 | **.006** | .059 |  | 1.79 | .183 | .014 |
| Message Format × Probability Rate | 15.17 | **<.001** | .108 |  | 26.33 | **<.001** | .174 |  | 12.91 | **<.001** | .094 |
| Tailoring × Probability Rate | < 1 | - | - |  | < 1 | - | - |  | < 1 | - | - |
| Tailoring × Message Format × Probability Rate | < 1 | - | - |  | < 1 | - | - |  | < 1 | - | - |

*Note.* ^a^ *df* = 1, 125; Significant results are given in **bold.**

**Table A2.** Main and interaction effects on primary outcome measures (i.e., risk estimates, risk accuracy, and risk perception), resulting from a 2 (tailoring: tailored, generic) × 2 (message format: verbal-only, verbal and numerical combined) × 2 (probability rate: low, high) MANCOVA with repeated measures on the first and third factor.

|  | Primary outcome variables | | | | | | | | | | |
| --- | --- | --- | --- | --- | --- | --- | --- | --- | --- | --- | --- |
|  | Estimation of probability | | |  | Accuracy of estimation of probability | | |  | Perceived likelihood of occurrence | | |
| Main/Interaction Effects | *F* | *p* | η_p_^2^ |  | *F* | *p* | η_p_^2^ |  | *F* | *p* | η_p_^2^ |
| Tailoring | 1.04 | .310 | .009 |  | 1.60 | .209 | .002 |  | 0.28 | .598 | .002 |
| Message Format | 63.43 | **<.001** | .348 |  | 59.28 | **<.001** | .333 |  | 27.13 | **<.001** | .186 |
| Probability Rate | 1.64 | .203 | .014 |  | 4.55 | **.035** | .037 |  | 2.03 | .157 | .017 |
| Tailoring × Message Format | 7.32 | **.008** | .058 |  | 6.70 | **.011** | .053 |  | 2.03 | .157 | .017 |
| Message Format × Probability Rate | 11.45 | **.001** | .088 |  | 21.14 | **<.001** | .151 |  | 9.00 | **.003** | .070 |
| Tailoring × Probability Rate | < 1 | - | - |  | < 1 | - | - |  | < 1 | - | - |
| Tailoring × Message Format × Probability Rate | < 1 | - | - |  | < 1 | - | - |  | < 1 | - | - |

*Note.* ^a^ *df* = 1, 125; ^b^ *df* = 1, 119; ^c^ Individual difference in numeracy, prior history with chemotherapy, and prior history with one of the side effects serve as covariates; Significant results are given in bold.
